# Psychoradiology: neuroimaging clinics of North America^☆^

**DOI:** 10.1093/psyrad/kkab006

**Published:** 2021-08-05

**Authors:** Kelvin Lim

**Affiliations:** Department of Psychiatry and Behavioral Sciences, University of Minnesota, 717 Delaware St. SE Suite 516, Minneapolis, MN 55403, USA

This volume is a collection of chapters on the topic of psychoradiology, an emerging subspecialty of radiology whose goal is to bring psychiatric imaging into clinical care in the service of diagnostics, prognostics, and treatment planning. The volume is edited by Dr Qiyong Gong, a pioneer of the field, who is a radiologist and Director of the Huaxi MR Research Center, Chengdu, Sichuan, China. Dr Gong and colleagues have proposed the “brain structure-function behavioral conjunction” theory in which brain structural alteration leads to clinical syndromes via impact on widely distributed functional connectivity. This theory provides a framework for identifying imaging biomarkers to specific psychiatric disorders. There are 11 chapters in total, written by 34 authors who are leaders in the field. The chapters can be divided into roughly three different categories: imaging modalities and image analysis, general approaches, and application to clinical disorders. The first chapter lays out the challenges and strategies for psychoradiology. This is followed by two chapters on two magnetic resonance methods: resting state functional connectivity and magnetic resonance spectroscopy. The following four chapters develop how radiology can be further applied to psychiatric disorders. There are chapters on the use of imaging for psychiatric subtyping, individual-specific analysis, biomarkers for psychopharmaceutical effects, and implementation of magnetic resonance imaging into routine clinical screening for patients with psychosis. The final four chapters are devoted to neuroimaging findings in clinical disorders. These disorders include schizophrenia, depression, autism, and posttraumatic stress disorder. The volume not only summarizes the developments and progress so far in psychoradiology, but also the challenges. There is practical information regarding the imaging examination needed, including imaging parameter and acquisition suggestions, the importance of standardization, and quality control.

This volume on psychoradiology is timely, as personalized medicine is becoming a reality in some fields of medicine such as cancer, but this is not yet the case for psychiatric disorders. The main reason for this is that we know far more about the molecular and pathophysiological mechanisms underlying cancer than we know about those underlying psychiatric disorders. Multimodality neuroimaging has been a critical tool in the study of psychiatric disorders. As described in the chapters on applications and clinical disorders, while there is evidence of statistically significant but subtle differences that can be detected between subjects with psychiatric disorder and healthy subjects, there is not yet robust enough information to make decisions at an individual level. As concluded by the authors, more work is needed that will require large samples coming from large-scale consortia with multimodal, noninvasive measures. So, while psychoradiology has not yet reached clinical utility, this volume captures the promise that multimodal psychoradiological approaches hold for advancing our understanding of psychiatric disorders and improving the clinical care of psychiatric patients.

